# Supplementary material for: KRASG12D and TP53R167H Cooperate to Induce Pancreatic Ductal Adenocarcinoma in Sus scrofa Pigs
Source: Sci Rep. 2018 Aug 22;8:12548. doi: 10.1038/s41598-018-30916-6 (PMC6105629; doi:10.1038/s41598-018-30916-6)
Supplement: Supplementary file 1 — Supplementary Information [file 41598_2018_30916_MOESM1_ESM.pdf]

# Supplement: KRAS$^{G12D}$ and TP53$^{R167H}$ Cooperate to Induce Pancreatic Ductal Adenocarcinoma in *Sus Scrofa* Pigs

Daniel R. Principe[1], Nana Haahr Overgaard[2,3], Alex J. Park[1], Andrew M. Diaz[4], Carolina Torres[4], Ronald McKinney[4], Matthew J. Dorman[4], Karla Castellanos[4], Regina Schwind[5], David W. Dawson[6], Ajay Rana[7], Ajay Maker[1], Hidayatullah G. Munshi[8], Laurie A. Rund[3,9], Paul J. Grippo[4*], and Lawrence B. Schook[3,9*]

[1]University of Illinois College of Medicine, Chicago, IL; [2]Department of Immunology and Vaccinology, National Veterinary Institute, Technical University of Denmark Copenhagen, Denmark; [3]University of Illinois Department of Animal Sciences, Urbana-Champaign, IL; [4]Department of Medicine, University of Illinois at Chicago, Chicago, IL; [5]University of Illinois Cancer Center, Chicago, IL; [6]Department of Pathology and Laboratory Medicine, Jonsson Comprehensive Cancer Center, David Geffen School of Medicine at UCLA, Los Angeles, California; [7]Department of Surgery, University of Illinois at Chicago, Chicago, IL. [8]Department of Medicine, Northwestern University, Chicago, IL, [9]Institute for Genomic Biology, University of Illinois, Urbana-Champaign, IL.

*Paul Grippo and Lawrence Schook contributed equally and serve as joint senior authors.

Short Title: KRAS$^{G12D}$ and TP53$^{R167H}$ Induce PDAC in Sus Scrofa Pigs

| | |
|---|---|
| Pages | 8 |
| Figures | 9 |
| Tables | 1 |
| Words | 1,742 |

[*] Correspondence to:

Paul J. Grippo, PhD
Associate Professor
Division of Gastroenterology and Hepatology
University of Illinois at Chicago
Department of Medicine
840 South Wood Street, 738A CSB
Chicago, IL 60612
pgrippo@uic.edu

or

Lawrence B. Schook, PhD
Director of the Division of Biomedical Sciences
1201 W Gregory Dr
382 ERML
Urbana, Illinois 61801
(217) 244-8480
schook@illinois.edu

## SUPPLEMENTARY RESULTS

### *Intra-Pancreatic Injection of Adeno-Cre in LSL-KRAS$^{G12D}$-TP53$^{R167H}$ Pigs Established Predominantly Metastastic Metastatic Leiomyosarcoma*

LSL-KRAS$^{G12D}$-TP53$^{R167H}$ pigs (N=3) were anesthetized, and 4x10$^{9}$ Adeno-Cre injected directly into the gland parenchyma (Figure S3A). Approximately two weeks following the surgery, the pigs began showing signs of pancreatic insufficiency including vomiting though there were no signs of jaundice/bile duct obstruction. Animals were euthanized 16 days following Adeno-Cre infection.

Gross necropsy revealed several abnormalities (summarized in Table S1) in 3/3 pigs, though these were particularly severe in one animal. This included 10-15 ml of a serosanguinous fluid in the abdominal cavity and 20-30ml of similar fluid in the thoracic cavity (data not shown). We also observed large abnormal masses surrounding the pancreatic duct and tapering toward the duodenum, which were continuous with the duodenal tunica muscularis. These masses were firmly adherent to the serosal surface of the duodenum, as well as multi-focally adherent to the omentum. On cut surface, the tumor appeared homogenous, firm and displayed extensive fibrosis. These masses were expanding into the pancreatic lobules, which were pale with noticeable hemorrhage and friable pale regions attributed to saponified fat (Figure S3A).

The squamous region of the stomach contained multiple, small erosions, and the omentum was thickened by numerous demarcated plaques (Figure S3A). Similar multifocal to coalescing plaques were observed on the splenic and hepatic capsules, with lesions being more pronounced on the lateral right lobe of the liver. Additionally, the abdominal surface of the diaphragm was granular and red, with plaques restricted only on the capsule and not invading the parenchyma (Figure S3B). Finally, blood was collected for complete blood count (CBC) and blood chemistry, both of which showed little to no

abnormality (data not shown).

To exclude the possibility that these masses were spontaneous or an indirect effect of the virus, the primary tumor was subject to RT-PCR, confirming the presence of the $TP53^{R167H}$ transcript (Figure S3C,D). Primary tumors were next sectioned, and staining for $RAS^{G12D}$ and $TP53^{R167H}$ affirmed their expression in malignant tissues and strongly localized with PCNA, a surrogate marker of cell proliferation (Figure S3E). However, consistent with our initial assessment, these malignant tissues were morphologically distinct from known pancreatic neoplasms, instead showed mesenchymal characteristics consistent with leimyosarcoma (Figure S3E). Furthermore, the peripancreatic lymph node was markedly enlarged in these animals (approximately 6-8 x the normal size) and lacked a distinct cortex/medulla, showing clear signs of metastasis consistent with the described sarcoma (Figure S3E). The peri-gastric and hepatic hilar lymph nodes were also enlarged, though they displayed distinct cortices and medullas (data not shown). The diagnosis of leiomyosarcoma was confirmed via immunohistochemistry. The malignant tissue was positive for mutant $KRAS^{G12D}$ and had strong expression of the KRAS effector pERK as well as proliferation surrogate PCNA (Figure S3F). This was not observed in the adjacent normal tissue, which had low expression of both pERK and PCNA (Figure S3F). Similarly, the infiltrating sarcoma was negative for the epithelial marker E-Cadherin, and highly positive for mesenchymal markers α-Smooth Muscle Actin (αSMA) and Vimentin, and displayed little of the fibrosis classically associated with PDAC (Figure S3F).

### ***Adeno-Cre Injected LSL-KRAS$^{G12D}$-TP53$^{R167H}$ Pigs Present with PanIN Disease with Several Hallmark Features of Pancreatic Tumorigenesis***

While leimyosarcoma was the predominant histotype observed and accounted for all masses observed upon necropsy, histological analysis of the pancreas revealed several large, luminal masses as well as pronounced abnormalities to the ductal tree. In each

animal, there were several lesions resembling a combination of acinar-to-ductal metaplasia (ADM) and pancreatic intraepithelial neoplasms (PanINs) (Figure S4A). While the majority of structures were well differentiated, they exhibited heterochromatic nuclei and there were isolated regions of metaplastic/neoplastic growth. These lesions were accompanied by extensive leukocyte infiltration and fibrosis, as well as the occasional intra-pancreatic hemorrhage in areas with invading leimyosarcoma (Figure S4A).

To affirm the PanIN lineage, tissues were stained for immunohistochemistry. Unlike the observed leiomyosarcoma, these lesions were highly positive for E-Cadherin indicating epithelial origin. Additionally, these lesions also exhibited strong PCNA staining suggesting increased cell proliferation and consistent with a PanIN phenotype. Additionally, these lesions were surrounded by a dense, desmoplastic stroma that stained positive for Masson's Trichrome as well as mesencyhmal markers Vimentin and αSMA, though the lesions themselves were negative for both Vimentin and αSMA (Figure S4B). Tissues were next stained for the duct marker CK19 and acinar cell marker Pancreatic Amylase. Consistent with previous observations, the invading sarcoma was negative for both makers. However, several of these lesions were dual positive for both CK19 and Pancreatic Amylase, confirming acinar-to-ductal metaplasia, an early event in pancreatic carcinogenesis (Figure S4C). Similarly, CK19-positive lesions displayed strong PCNA staining indicative of cell proliferation, and were surrounded by a αSMA-rich tumor stroma (Figure S4C).

## SUPPLEMENTARY FIGURE LEGENDS

**Figure S1. Comparative histology between human, mouse, and pig pancreas.**

Human, mouse, and pig pancreata were sectioned and H&E stained to assess structural similarities in **(A)** pancreatic acini, **(B)** pancreatic ductal system, and **(C)** neuroendocrine Islets of Langerhans.

**Figure S2. Adeno-Cre induces expression of mutant KRAS$^{G12D}$ and P53$^{R167H}$ proteins, and confers increased KRAS activity**

**(A)** Control and Adeno-Cre transformed duct cells LSL-KRAS$^{G12D}$-TP53$^{R167H}$ pigs were evaluated by western blotting for expression of mutant RAS (mtRAS), mutant P53 (mtP53), and downstream ERK activation. **(B)** Lysates were subject to a KRAS activity assay, indicating increased KRAS-GTP association in Adeno-Cre transformed duct cells.

**Figure S3. PORC1 cells establish subcutaneous tumors in immunocompromised mice**

**(A)** 5-10x10$^6$ PORC1 cells were injected intraperitoneally into SCID mice. After 6, the mice developed palpable tumors at the site of injection. After 12 days, animals were euthanized and tumors sectioned/stained with H&E, Mason's Trichrome, or immunohistochemistry stained for mutant P53 (mtP53), E-cadherin, PCNA, and RAS$^{G12D}$. **(B)** Animal health was monitored by tracking changes in weight, and tumor growth by carefully measuring the tumor size.

**Figure S4. Intra-pancreatic injection of Adeno-Cre in LSL-KRAS$^{G12D}$-TP53$^{R167H}$ pigs induces predominantly metastastic leiomyosarcoma**

**(A)** Approximately 4-6 month old LSL-KRAS$^{G12D}$-TP53$^{R167H}$ pigs (N=3) were anesthetized and 4x10$^9$ Adeno-Cre injected into pancreas gland. 16 days later, pigs developed large masses surrounding the pancreatic duct and tapering toward the duodenum, which were adherent to the greater omentum (G.O.). Pancreatic epithelial tissue is denoted by the white box and shown at higher magnification in the bottom right panel. **(B)** Multifocal to

coalescing plaques were observed on the splenic and hepatic capsules and are denoted with white arrows. **(C,D)** Tumors from the liver/spleen were evaluated by RT-PCR for the $TP53^{R167H}$ transcript. **(E)** Primary tumors were sectioned and stained with H&E, showing morphology distinct from known pancreatic neoplasms, with more mesenchymal characteristics consistent with leiomyosarcoma. Histology also confirmed the presence of similar cells in the tunica muscularis of the duodenum and regional lymph nodes. **(F)** Tissue sections were stained via immunohistochemistry for mutant $RAS^{G12D}$, pERK, and PCNA, as well as differentiation markers E-cadherin, αSMA, and Vimentin. Fibrosis was assessed via Masson's Trichrome staining.

**Figure S5. Adeno-Cre Injected LSL-$KRAS^{G12D}$-$TP53^{R167H}$ Pigs Present with PanIN Disease with Several Hallmark features of Pancreatic Tumorigenesis**

**(A)** Primary tumors were sectioned and stained with H&E. While the dominant histoype was indeed leiomyosarcoma, there were several large, luminal masses throughout the pancreas consistent with mucinous, PanIN-like pancreatic neoplasms. **(B)** These PanINs were assessed via immunohistochemistry and were positive for epithelial marker E-cadherin and proliferation surrogate PCNA, with Mason's Trichrome, vVimetnin, and αSMA staining confined to the stroma surrounding the lesions. **(C)** To assess acinar-to-ductal metaplasia (ADM), a hallmark of early pancreatic tumorigenesis, sections were dual-stained for duct marker CK19 and acinar marker Pancreatic Amylase. In addition, co-staining for CK19/PCNA and CK19/αSMA was also performed.

**Figure S6. Restriction of Adeno-Cre to the Main Pancreatic Duct Induces Tumors Along the Injection Site**

**(A)** $4x10^9$ Adeno-Cre was injected directly into the main pancreatic duct of the LSL-$KRAS^{G12D}$-$TP53^{R167H}$ pig. One year after the injection, pancreas of was evaluated by CT

imaging. **(B)** Pigs were euthanized, the pancreas evaluated for tumor development by gross histology and by H&E staining.

**Figure S7. Intraductal Delivery of Ad-Cre Induces Areas of Neuroendocrine Carcinoma**

**(A)** Sections from human patients with pancreatic neuroendocrine tumors were stained with H&E or trichrome and compared to **(B)** tumors from the LSL-KRAS$^{G12D}$-TP53$^{R167H}$ pig delivered an Adeno-Cre injection into the main pancreatic duct suspect **(C,D)** Porcine tumors were next stained via immunohistochemistry for the neuroendocrine marker synaptophysin, as well the RAS target pERK or proliferation surrogate PCNA.

**Figure S8. Porcine tumors express mutant KRAS$^{G12D}$ and P53$^{R167H}$ and display increased KRAS activity**

**(A)** Control (Ctrl) and Tumor (Tu) tissue was evaluated by western blotting for stromal markers Collagen IA (COLIA) and Vimetin, as well as for expression of mutant RAS (mtRAS), mutant P53 (mtP53), and downstream ERK activation. **(B)** Lysates from control and tumor tissue were subject to a KRAS activity assay, indicating increased KRAS-GTP association in Adeno-Cre transformed duct cells.

**Figure S9. Intraductal delivery of Ad-Cre increases ERK activation and cell proliferation**

Tumors from Adeno-Cre injected LSL-KRAS$^{G12D}$-TP53$^{R167H}$ pigs were sectioned and stained for pERK or PCNA. Cells positive for either **(A)** pERK or **(B)** PCNA were quanitified per 40X field by two blinded investigators. Counts were averaged and displayed as mean ± SEM (*$P < 0.05$).

## Table S1

| | |
|---|---|
| **Pancreas** | Leimyosarcoma with: severe necrotizing and lymphoplasmacytic pancreatitis with fibrosis, acinar degeneration, acinar-to-ductal metaplasia, severe necrotizing peripancreatic steatitis, isolated endocrine/exocrine cell necrosis, and fibrosis/inflammaton associated with the main pancreatic duct. Tumor is mostly centered and surrounds the common arm of pancreatic duct and pancreas. |
| **Small Intestine** | Neoplastic cells similar to those described previously are present in the duodenum. |
| **Liver** | Secondary tumors consistent with that described previously. Portal triads are infiltrated by small numbers of lymphocytes and plasma cells. Neoplastic cells similar to those described previously are present on the capsule and are invading the underlying subcapsular parenchyma. |
| **Bile Duct** | Neoplastic cells similar to those described previously are present in the periductal tissue. |
| **Lymph Nodes** | Peripancreatic lymph node 6-8x normal size and lacking a distinct cortex and medulla and showing clear signs of metastasis. Perigastric and hepatic hilar lymph nodes also enlarged with no evidence of metastases. |
| **Spleen** | Multifocal to coalescing plaques were observed on the splenic capsule consistent with metastases. |
| **Omentum** | Neoplastic cells similar to those described previously are present. |
| **Colon** | No Significant Lesion (NSL), moderate lymphocytes/plasma cells and rare eosinophils present within the lamina propria. |
| **Thyroid** | NSL |
| **Lung** | NSL, rare peribronchial aggregates of lymphocytes and plasma cells. |
| **Heart** | NSL |
| **Kidneys** | Cortex and medulla are infiltrated by small aggregates of lymphocytes and plasma cells. Thickened glomerular mesangium. |
| **Diaphragm** | Granular and red with focal lesions consistent with the previously descried neoplasms. |
| **Esophagus** | NSL |
| **Bladder** | NSL |
| **Stomach** | NSL, multiple, small erosions. |

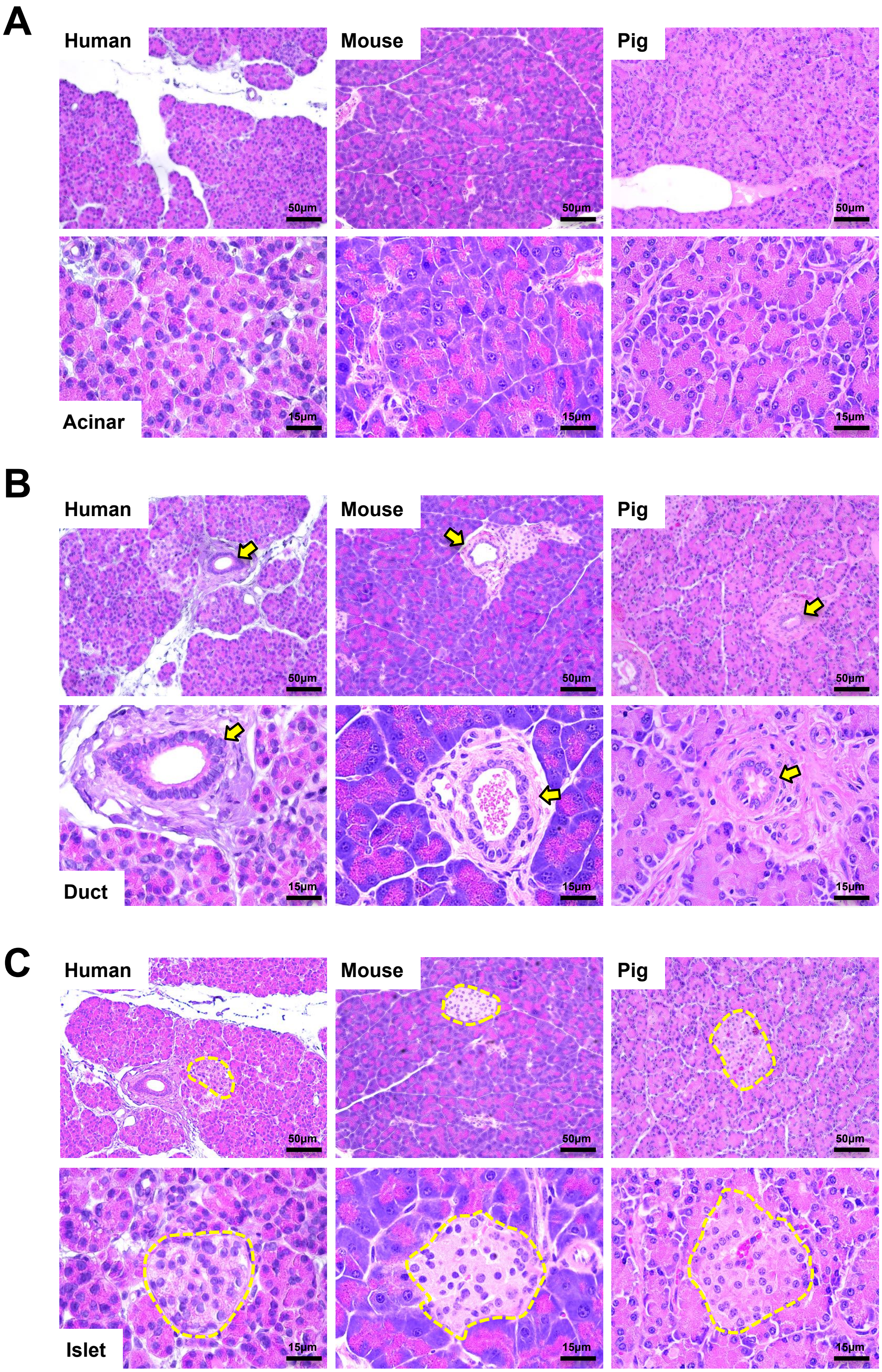


**Figure S1. Comparative histology between human, mouse, and pig pancreas**

Human, mouse, and pig pancreata were sectioned and H&E stained to assess structural similarities in **(A)** pancreatic acini, **(B)** pancreatic ductal system, and **(C)** neuroendocrine Islets of Langerhans.

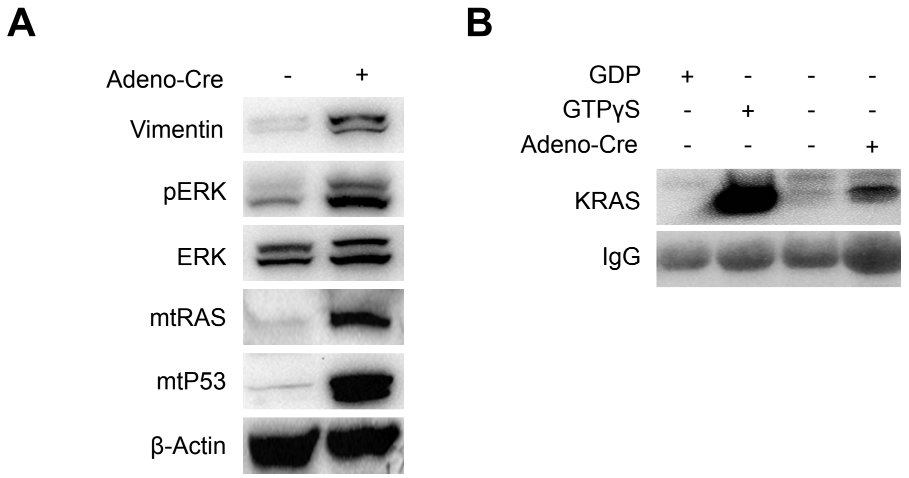


**Figure S2. Adeno-Cre induces expression of mutant KRAS$^{G12D}$ and P53$^{R167H}$ proteins, and confers increased KRAS activity**

**(A)** Control and Adeno-Cre transformed duct cells LSL-KRAS$^{G12D}$-TP53$^{R167H}$ pigs were evaluated by western blotting for expression of mutant RAS (mtRAS), mutant P53 (mtP53), and downstream ERK activation. **(B)** Lysates were subject to a KRAS activity assay, indicating increased KRAS-GTP association in Adeno-Cre transformed duct cells.

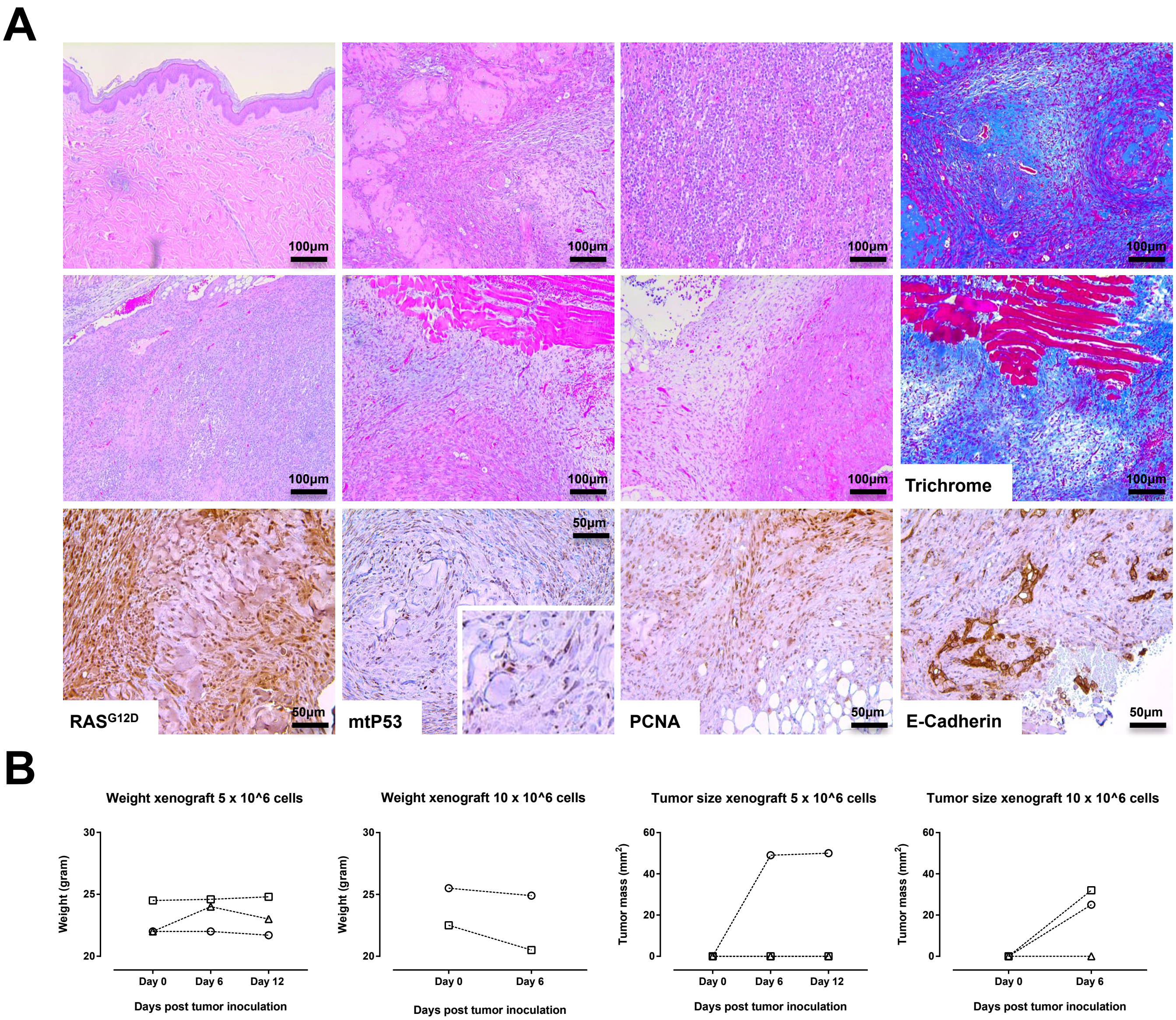


**Figure S3. PORC1 cells establish subcutaneous tumors in immunocompromised mice**

**(A)** 5-10x10$^6$ PORC1 cells were injected intraperitoneally into SCID mice. After 6, the mice developed palpable tumors at the site of injection. After 12 days, animals were euthanized and tumors sectioned/stained with H&E, Mason's Trichrome, or immunohistochemistry stained for mutant P53 (mtP53), E-cadherin, PCNA, and RAS$^{G12D}$. **(B)** Animal health was monitored by tracking changes in weight, and tumor growth by carefully measuring the tumor size.

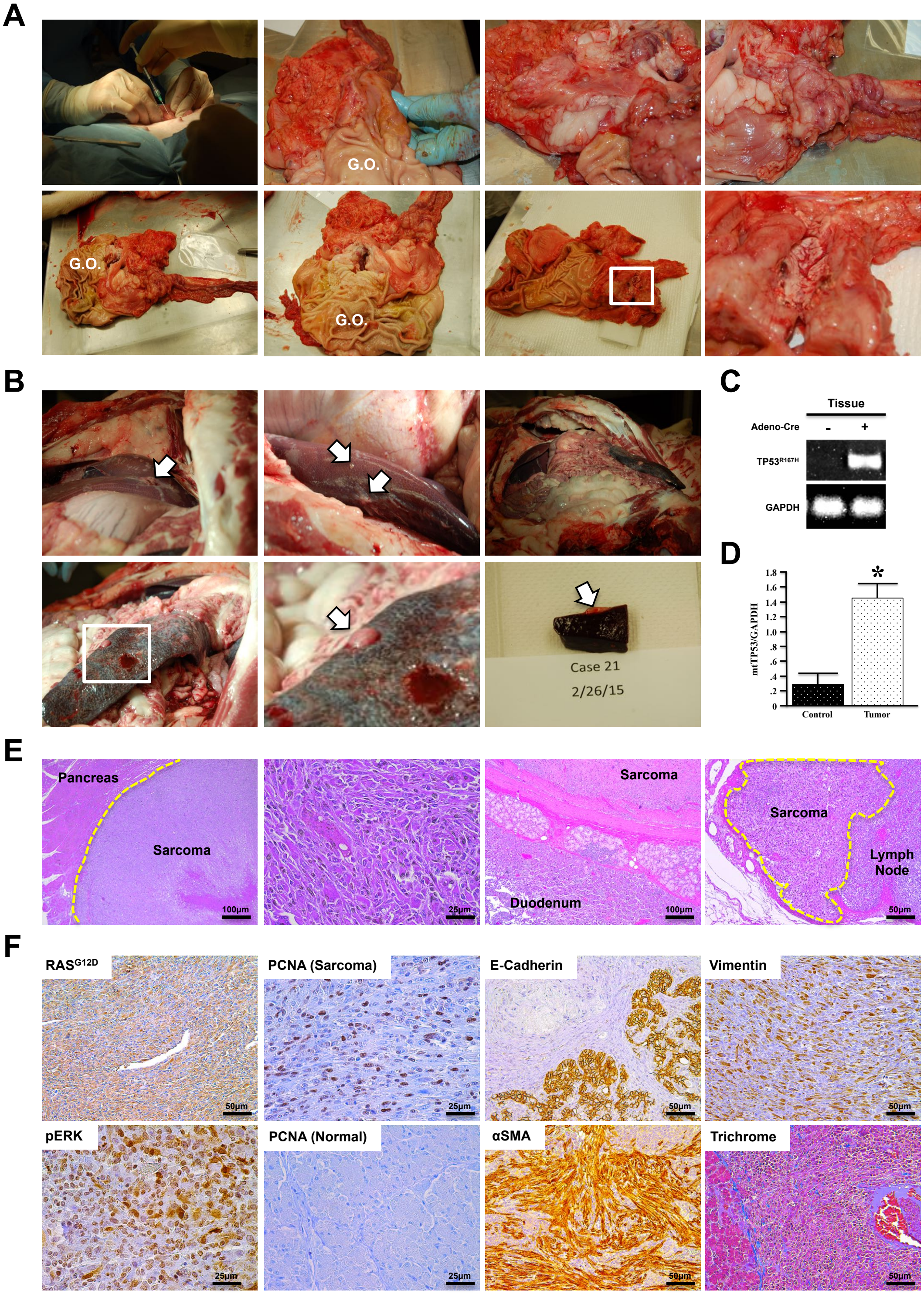


**Figure S4. Intra-pancreatic injection of Adeno-Cre in LSL-KRAS$^{G12D}$-TP53$^{R167H}$ pigs induces predominantly metastastic leiomyosarcoma**

**(A)** Approximately 4-6 month old LSL-KRAS$^{G12D}$-TP53$^{R167H}$ pigs (N=3) were anesthetized and 4x10$^{9}$ Adeno-Cre injected into pancreas gland. 16 days later, pigs developed large masses surrounding the pancreatic duct and tapering toward the duodenum, which were adherent to the greater omentum (G.O.). Pancreatic epithelial tissue is denoted by the white box and shown at higher magnification in the bottom right panel. **(B)** Multifocal to coalescing plaques were observed on the splenic and hepatic capsules and are denoted with white arrows. **(C,D)** Tumors from the liver/spleen were evaluated by RT-PCR for the TP53$^{R167H}$ transcript. **(E)** Primary tumors were sectioned and stained with H&E, showing morphology distinct from known pancreatic neoplasms, with more mesenchymal characteristics consistent with leiomyosarcoma. Histology also confirmed the presence of similar cells in the tunica muscularis of the duodenum and regional lymph nodes. **(F)** Tissue sections were stained via immunohistochemistry for mutant RAS$^{G12D}$, pERK, and PCNA, as well as differentiation markers E-cadherin, αSMA, and Vimentin. Fibrosis was assessed via Masson's Trichrome staining.

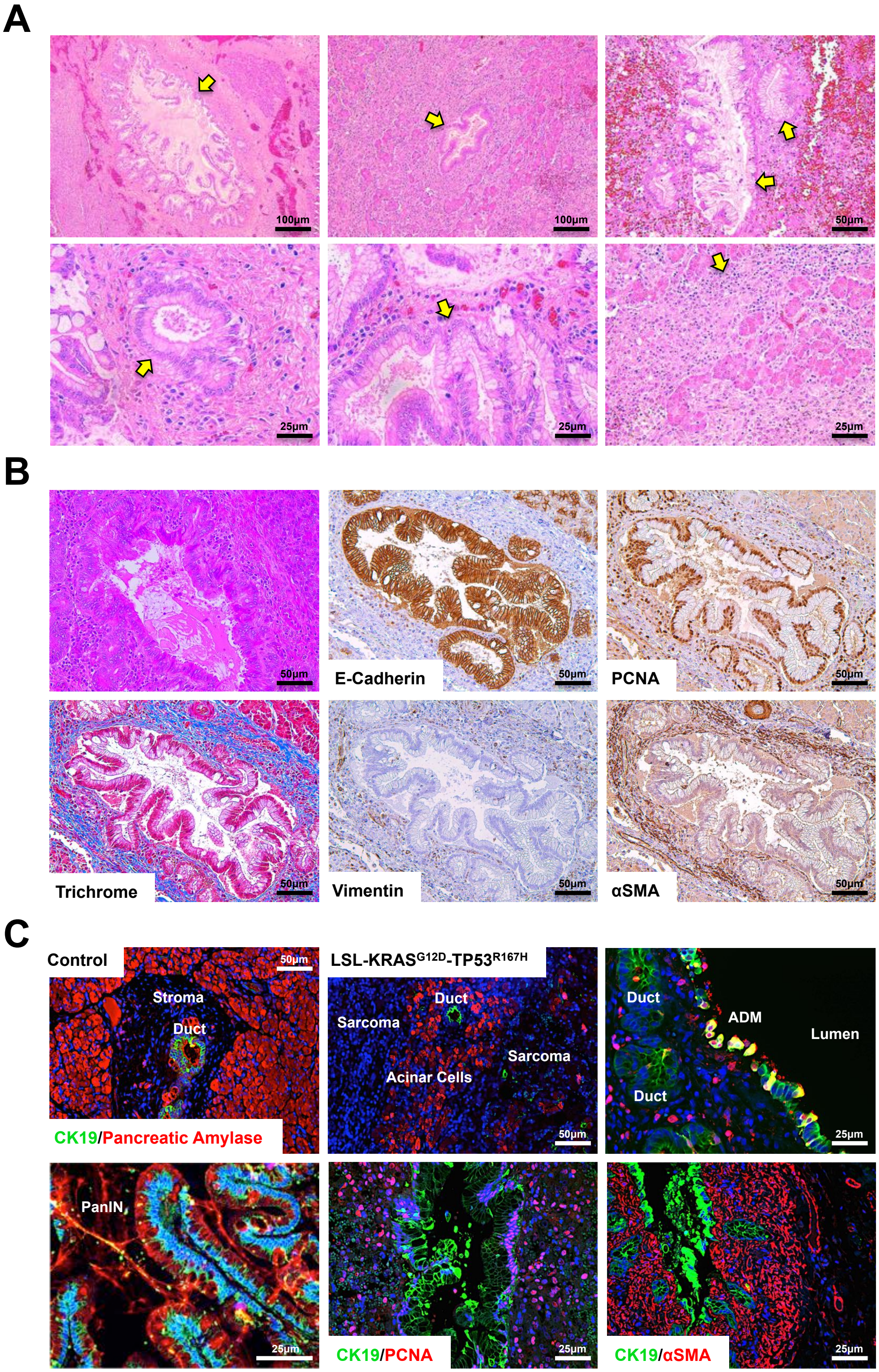


**Figure S5. Adeno-Cre injected LSL-KRAS$^{G12D}$-TP53$^{R167H}$ pigs present with PanIN disease with several hallmark features of pancreatic tumorigenesis**

**(A)** Primary tumors were sectioned and stained with H&E. While the dominant histoype was indeed leiomyosarcoma, there were several large, luminal masses throughout the pancreas consistent with mucinous, PanIN-like pancreatic neoplasms. **(B)** These PanINs were assessed via immunohistochemistry and were positive for epithelial marker E-cadherin and proliferation surrogate PCNA, with Masson's Trichrome, vVimetnin, and αSMA staining confined to the stroma surrounding the lesions. **(C)** To assess acinar-to-ductal metaplasia (ADM), a hallmark of early pancreatic tumorigenesis, sections were dual-stained for duct marker CK19 and acinar marker Pancreatic Amylase. In addition, co-staining for CK19/PCNA and CK19/αSMA was also performed.

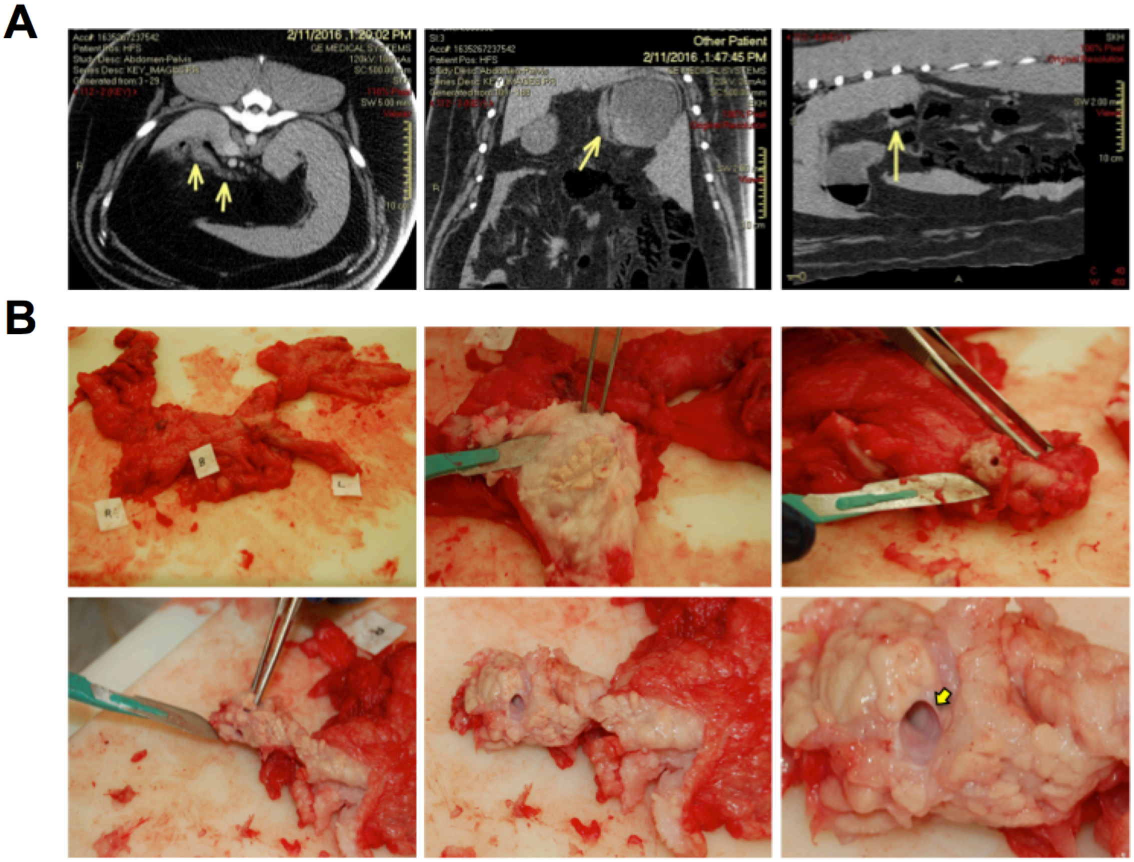


**Figure S6. Restriction of Adeno-Cre to the main pancreatic duct induces tumors along the injection site**

**(A)** $4x10^9$ Adeno-Cre was injected directly into the main pancreatic duct of the LSL-KRAS$^{G12D}$-TP53$^{R167H}$ pig. One year after the injection, pancreas of was evaluated by CT imaging. **(B)** Pigs were euthanized, the pancreas evaluated for tumor development by gross histology and by H&E staining.

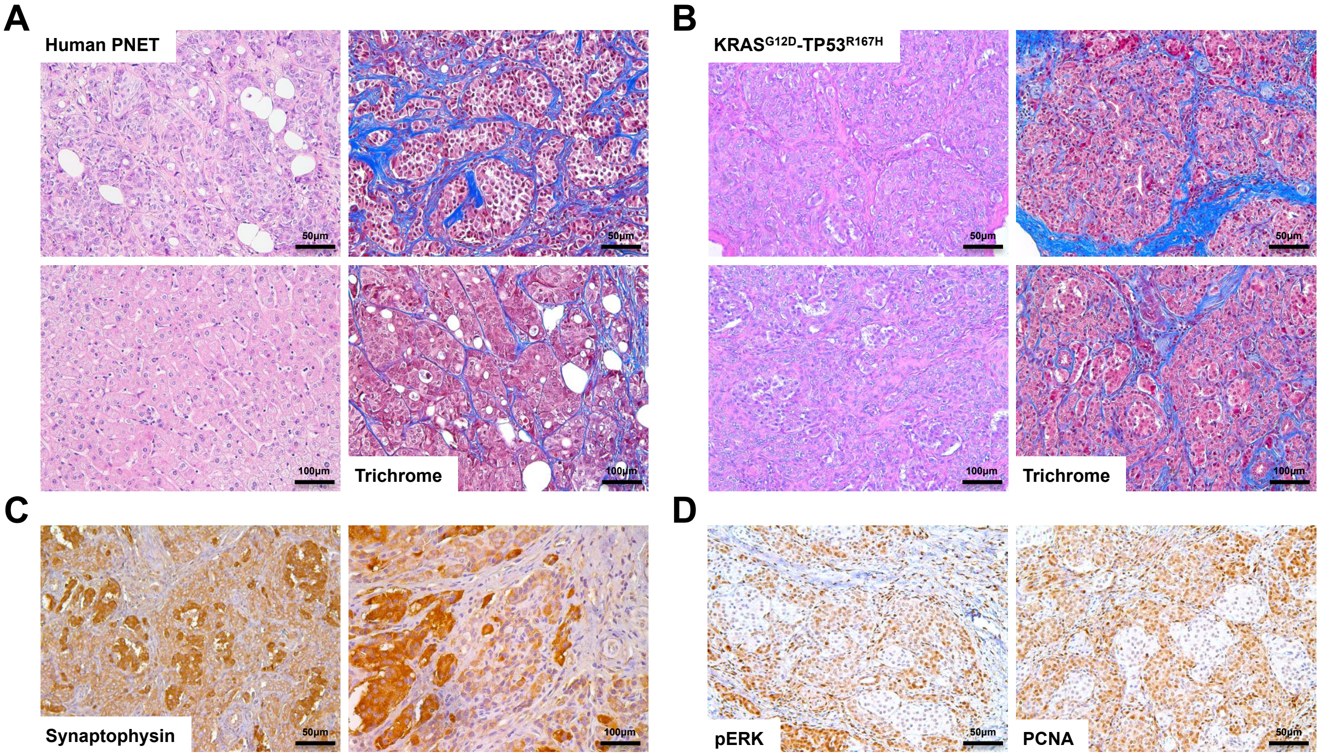


**Figure S7. Intraductal delivery of Ad-Cre induces areas of neuroendocrine carcinoma**

**(A)** Sections from human patients with pancreatic neuroendocrine tumors were stained with H&E or trichrome and compared to **(B)** tumors from the LSL-KRAS$^{G12D}$-TP53$^{R167H}$ pig delivered an Adeno-Cre injection into the main pancreatic duct suspect **(C,D)** Porcine tumors were next stained via immunohistochemistry for the neuroendocrine marker Synaptophysin, as well the RAS target pERK or proliferation surrogate PCNA.

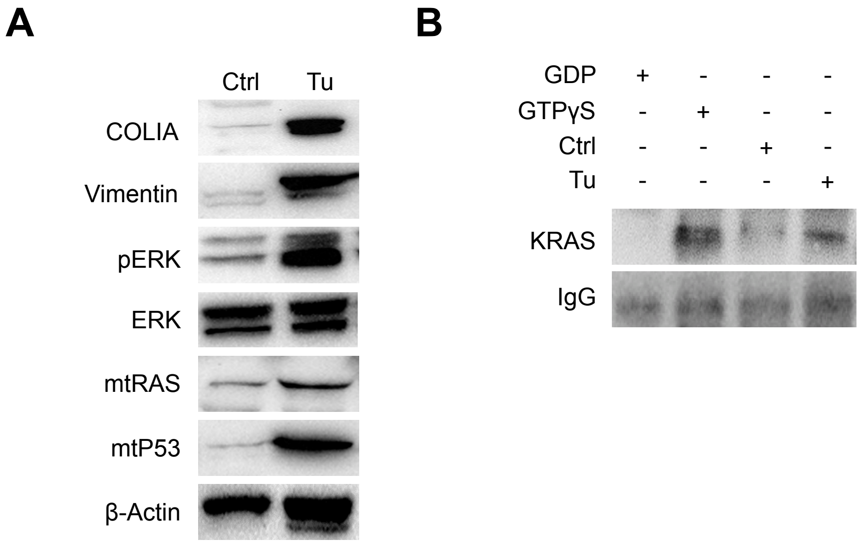


**Figure S8. Porcine tumors express mutant KRAS$^{G12D}$ and P53$^{R167H}$ and display increased KRAS activity**

**(A)** Control (Ctrl) and Tumor (Tu) tissue was evaluated by western blotting for stromal markers Collagen IA (COLIA) and Vimetin, as well as for expression of mutant RAS (mtRAS), mutant P53 (mtP53), and downstream ERK activation. **(B)** Lysates from control and tumor tissue were subject to a KRAS activity assay, indicating increased KRAS-GTP association in Adeno-Cre transformed duct cells.

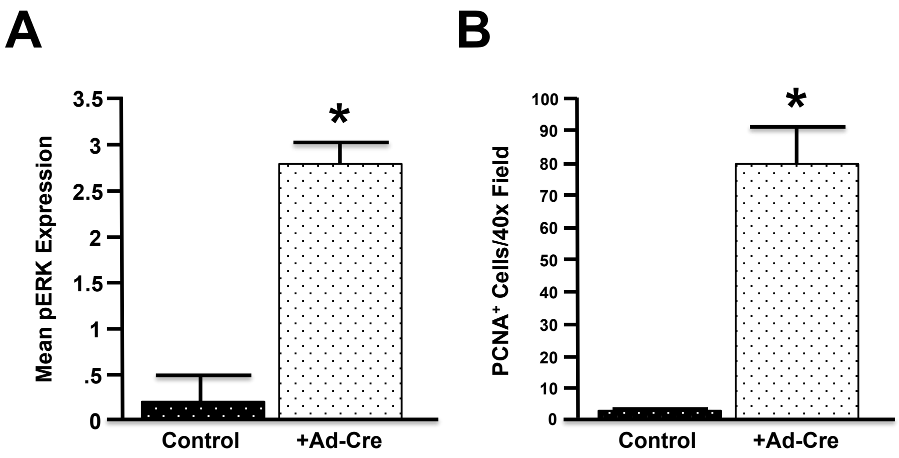


**Figure S9. Intraductal delivery of Ad-Cre increases ERK activation and cell proliferation**

Tumors from Adeno-Cre injected LSL-KRAS$^{G12D}$-TP53$^{R167H}$ pigs were sectioned and stained for pERK or PCNA. Cells positive for either **(A)** pERK or **(B)** PCNA were quanitified per 40X field by two blinded investigators. Counts were averaged and displayed as mean SEM (*P < 0.05)

| | |
|---|---|
| **Pancreas** | Leimyosarcoma with: severe necrotizing and lymphoplasmacytic pancreatitis with fibrosis, acinar degeneration, acinar-to-ductal metaplasia, severe necrotizing peripancreatic steatitis, isolated endocrine/exocrine cell necrosis, and fibrosis/inflammaton associated with the main pancreatic duct. Tumor is mostly centered and surrounds the common arm of pancreatic duct and pancreas. |
| **Small Intestine** | Neoplastic cells similar to those described previously are present in the duodenum. |
| **Liver** | Secondary tumors consistent with that described previously. Portal triads are infiltrated by small numbers of lymphocytes and plasma cells. Neoplastic cells similar to those described previously are present on the capsule and are invading the underlying subcapsular parenchyma. |
| **Bile Duct** | Neoplastic cells similar to those described previously are present in the periductal tissue. |
| **Lymph Nodes** | Peripancreatic lymph node 6-8x normal size and lacking a distinct cortex and medulla and showing clear signs of metastasis. Perigastric and hepatic hilar lymph nodes also enlarged with no evidence of metastases. |
| **Spleen** | Multifocal to coalescing plaques were observed on the splenic capsule consistent with metastases. |
| **Omentum** | Neoplastic cells similar to those described previously are present. |
| **Colon** | No Significant Lesion (NSL), moderate lymphocytes/plasma cells and rare eosinophils present within the lamina propria. |
| **Thyroid** | NSL |
| **Lung** | NSL, rare peribronchial aggregates of lymphocytes and plasma cells. |
| **Heart** | NSL |
| **Kidneys** | Cortex and medulla are infiltrated by small aggregates of lymphocytes and plasma cells. Thickened glomerular mesangium. |
| **Diaphragm** | Granular and red with focal lesions consistent with the previously descried neoplasms. |
| **Esophagus** | NSL |
| **Bladder** | NSL |
| **Stomach** | NSL, multiple, small erosions. |

**Table S1. Necropsy report from Intra-pancreatic injection of Adeno-Cre into LSL-KRAS$^{G12D}$-TP53$^{R167H}$ pigs**
